# Optimized enzymatic dual functions of PaPrx protein by proton irradiation

**DOI:** 10.1093/jrr/rrt081

**Published:** 2013-06-09

**Authors:** Chul-Hong Park, Seung Sik Lee, Kye Ryung Kim, Myung Hwan Jung, Sang Yeol Lee, Eun Ju Cho, Sudhir Singh, Byung Yeoup Chung

**Affiliations:** 1 Advanced Radiation Technology Institute, Korea Atomic Energy Research Institute, Jeongeup 580-185, Republic of Korea; 2 Proton Engineering Frontier Project, Korea Atomic Energy Research Institute, Gyeongju 780-904, Republic of Korea; 3 Division of Applied Life Sciences (BK21 Program), Gyeongsang National University, Jinju 660-701, Republic of Korea; 4 School of Biological Sciences and Biotechnology, Chonnam National University, Gwangju 500-757, Republic of Korea

**Keywords:** chaperone, peroxidase, peroxiredoxin, proton irradiation

## Abstract

We investigated the effects of proton irradiation on the function and structure of the *Pseudomonas aeruginosa* peroxiredoxin (PaPrx). Polyacrylamide gel demonstrated that PaPrx proteins exposed to proton irradiation at several doses exhibited simultaneous formation of high molecular weight (HMW) complexes and fragmentation. Size-exclusion chromatography (SEC) analysis revealed that the number of fragments and very low molecular weight (LMW) structures increased as the proton irradiation dose increased. The peroxidase activity of irradiated PaPrx was preserved, and its chaperone activity was significantly increased by increasing the proton irradiation dose. The chaperone activity increased about 3–4 fold after 2.5 kGy proton irradiation, compared with that of non-irradiated PaPrx, and increased to almost the maximum activity after 10 kGy proton irradiation. We previously obtained functional switching in PaPrx proteins, by using gamma rays and electron beams as radiation sources, and found that the proteins exhibited increased chaperone activity but decreased peroxidase activity. Interestingly, in this study we newly found that proton irradiation could enhance both peroxidase and chaperone activities. Therefore, we can suggest proton irradiation as a novel protocol for conserved 2-Cys protein engineering.

## INTRODUCTION

Peroxiredoxins (Prxs) are present in all living organisms from bacteria to mammals [1]. Prxs exert their protective antioxidant role in cells through their peroxidase activity, whereby hydrogen peroxide, peroxynitrite and a wide range of organic hydroperoxides (ROOH) are reduced and detoxified [2]. During the reaction cycle, the peroxidatic cysteine (Cys) residue is oxidized to sulfenic acid, whereas hydrogen peroxide, peroxynitrite, and a broad range of alkyl hydroperoxides are reduced to water, nitrite, or the corresponding alcohol [3–5]. Most Prxs belong to the 2-Cys Prx group and contain two conserved Cys residues that participate in catalysis. The Prxs, such as molecular chaperone protein, recognize and bind nascent polypeptide chains and partially folded protein intermediates, preventing their aggregation and misfolding [6]. The oligomerization of Prx leads to an increase in surface hydrophobicity, which allows chaperone activity to be increased [7].

Many recent studies have demonstrated that Prxs exhibit dual physiological functions, acting as both a peroxidase and a molecular chaperone [8–10]. The molecular chaperone activity has received considerable attention in recent years as a new role for these thiol-specific antioxidant proteins [8, 11–14]. The Prx protein structure generally changes from a low molecular weight (LMW) form to high molecular weight (HMW) complexes upon exposure to oxidative stress, heat shock, or phosphorylation, and enzymatic function is also reversibly switched from peroxidase activity to molecular chaperone activity [8, 12, 15, 16]. For example, under heat stress, the heat shock protein Hsp33 undergoes conformational changes that allow the formation of oxidized dimers of Hsp33. The oxidative form of these dimers shows highly efficient chaperone activity, whereas, under reducing conditions, Hsp33 monomers are inactive as chaperones [16]. Furthermore, the hPrx I, which contains a consensus site (Thr90-Pro-Lys-Lys) for phosphorylation by CDK1, is phosphorylated specifically on Thr90 (T90D-hPrx I), and T90D-hPrx I exhibited markedly decreased peroxidase activity and about six times the chaperone activity because its structural changes caused oligomer association [15].

Previously, we reported the effects of gamma rays or electron beams on the dual functions of Prxs [17, 18]. The chaperone activity of *Pseudomonas putida* peroxiredoxin (PpPrx) and *P. aeruginosa* peroxiredoxin (PaPrx) increased significantly (about 3–4-fold), but the peroxidase activity decreased with increasing radiation dose. Radiation of the protein causes the following chemical changes: fragmentation, cross-linking, aggregation, and oxidation by oxygen radicals that are generated in the radiolysis of water [19–23]. Although our earlier work suggested that the structural changes increased the chaperone activity and may be an important mechanism of functional switching in Prxs, gamma-ray and electron-beam irradiation decreased the peroxidase activity of these Prxs [17, 18]. To overcome this undesirable effect, we propose a novel method of increasing or optimizing the dual functions of Prxs by proton irradiation as an excellent alternative.

In this study, we investigate the effect of proton irradiation of PaPrx on its enzymatic dual functions and the optimal intensity of the irradiation. In addition, to explain the increased dual functions of modified PaPrx, we analyze the protein's hydrophobicity, size, and secondary structural changes as related to peroxidase and chaperone activity.

## MATERIALS AND METHODS

### Bacterial strains, media, and materials

The bacterial strains *P. aeruginosa* PAO1 and *Escherichia coli* DH5α (Promega, WI, USA), KRX (Promega) were grown aerobically at 30°C and 37°C, respectively, in Luria-Bertani (LB) medium (DB, NJ, USA) and were used to clone the *Prx* gene. Yeast thioredoxin (Trx) and thioredoxin reductase (TR) were prepared as described elsewhere [24]. The protein molecular size standards used in polyacrylamide gel electrophoresis (PAGE) were purchased from ELPIS (ELPIS, Daejeon, Korea). Ampicillin (Amp), imidazole, L-rhamnose, bovine serum albumin (BSA), hydrogen peroxide (H_2_O_2_; 30% v/v), and nicotinamide adenine dinucleotide phosphate (NADPH) were obtained from Sigma (Sigma, MO, USA).

### Cloning of *PaPrx* gene and expression in *E. coli*

The *PaPrx* gene was cloned from *P. aeruginosa* PAO1 genomic DNA by polymerase chain reaction (PCR). Briefly, PCR reactions were conducted in 20 µl mixtures containing 10 ng of genomic DNA, 0.2 µM deoxyribonucleotide triphosphates, 20 pmol of each primer set for *PaPrx* (forward primer: 5′-ccgctcgagatgagcgtactcgta, reverse primer: 3′-cccaagcttttacagcttgctggc), and 1 unit of Taq DNA polymerase (Promega) in a standard PCR buffer. The following conditions were used for PCR: denaturation for 1 cycle at 94°C for 60 s; 35 cycles each at 94°C for 30 s, at 50°C for 45 s, and at 72°C for 45 s; and finally 1 cycle at 72°C for 10 min. The PCR products were collected and purified by agarose gel electrophoresis. They were subcloned into the pGEM-T vector to produce *PaPrx*, which was then transformed into *E. coli* DH5α cells. These constructs were confirmed first by nucleotide sequencing and then by digesting the *PaPrx* fragment from pGEM-T by restriction enzymes (*Hind* III and *Xho* I). The prepared *PaPrx* fragment was subcloned into the *pRSETa* expression vector containing a His_6_ tag to create *pRSETa::PaPrx*. The His_6_-fused *PaPrx* was purified by using a Ni^2+^-nitrilotriacetate-agarose column (Peptron, Daejeon, Korea) and eluted with 150 mM imidazole in phosphate-buffered saline buffer [25]. After dialysis against 50 mM Tris-HCl buffer (pH 7.5), the protein concentration was measured using the Bradford method [26] with BSA as the standard.

### Proton irradiation

**Fig. 1.**
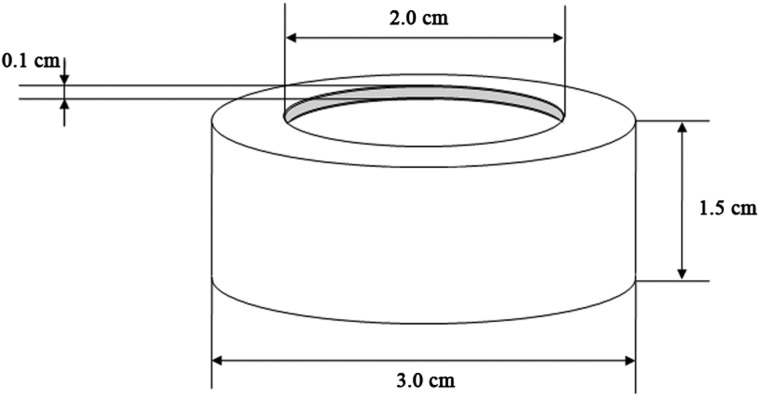
Acrylic vessel for proton irradiation.

Proton irradiation was performed using a 20-MeV proton linear accelerator installed at the Korea Atomic Energy Research Institute (Daejeon, Korea). The energy of protons extracted from the accelerator was reduced from 20 MeV to 14.8 MeV by passing them through a 0.5-mm-thick Al beam window and then through 70 cm of air. The result of a Stopping and Range of Ions in Matter (SRIM) code simulation from http://www.srim.org indicated that the 14.8 MeV protons that arrived at the surface of the sample can penetrate at most a 2.42-mm-thickness of water. Considering the beam profile and calculated penetration depth, we chose 1 mm and 2 cm for the thickness and diameter of the sectional area of the sample vessel, respectively. PaPrx proteins (2 mg/ml) were individually divided into 300–330 µl aliquots in dedicated acrylic vessels for proton irradiation (Fig. 1). Sample vessels were covered by 75-μm-thick polyimide films and were irradiated at 1–10 kGy and an average dose rate of 138.9 Gy/s at room temperature.

### Peroxidase and chaperone activity assay

The Trx-dependent peroxidase activity of the purified PaPrx was measured as the decrease in absorbance at 340 nm caused by NADPH oxidation [27, 28]. The PpPrx derivative protein was incubated in 50 mM HEPES (pH 8.0) containing 200 µM NADPH, 3 µM yeast Trx and 1.5 µM yeast TR. The reaction mixture was incubated at 30°C for 5 min, and then 10 µl of 50 mM H_2_O_2_ (final 1 mM) was added. NADPH oxidation was monitored for the next 15 min by measuring the decrease in A_340_ using an EVOLUTION 300 UV-VIS spectrophotometer (Thermoscientific, WI, USA). The chaperone activity was determined as described previously by assessing the ability of recombinant Prxs to inhibit the thermal aggregation of substrate proteins [12, 25, 27–29]. Briefly, 1 µM malate dehydrogenase (MDH) was mixed with 1 µM of non-irradiated and irradiated PaPrx proteins in a degassed 50 mM HEPES solution (pH 8.0). The reaction mixture was measured at 43°C for 15 min. The increase in light scattering as a result of thermal aggregation of the substrate protein was monitored at 340 nm with an EVOLUTION 300 UV-VIS spectrophotometer equipped with a thermostatic cell holder (Thermoscientific, WI, USA).

### Size-exclusion chromatography

Size-exclusion chromatography (SEC) was performed at 4°C by fast protein liquid chromatography (Amersham Biosciences, Uppsala, Sweden) using a Superdex 200 10/300 GL column. The PaPrx and irradiated PaPrx were injected into the column, which was equilibrated with 50 mM HEPES buffer (pH 8.0) containing 100 mM NaCl at a flow rate of 0.5 ml/min at 4°C. The area under the protein peaks (A_280_) was divided into three sections representing HMW complexes, LMW structures and fragments, respectively, and the area of each section was calculated in proportion to the total area [25].

### Circular dichroism spectroscopy

Far-UV circular dichroism (CD) spectra were used to investigate the changes in secondary structural elements due to proton irradiation. PaPrx and irradiated PaPrx proteins in 10 mM Tris-HCl buffer (pH 7.4) were used for Far UV-CD spectral analysis with a Jasco J-715 spectropolarimeter (Jasco, GD, UK), as described elsewhere [30].

### Fluorescence measurement

The hydrophobicity of PaPrx and its irradiated proteins was monitored by an Infinite M200 spectrofluorometer (Tecan, NC, USA) to examine the binding of 40 µM bis-ANS to 30 µg of each of the PaPrx proteins. Spectra were accumulated five times. The excitation wavelength was set to 380 nm, and emission spectra were monitored from 400–600 nm [31].

## RESULTS

### Effects of proton irradiation on the dual functions of PaPrx

**Fig. 2.**
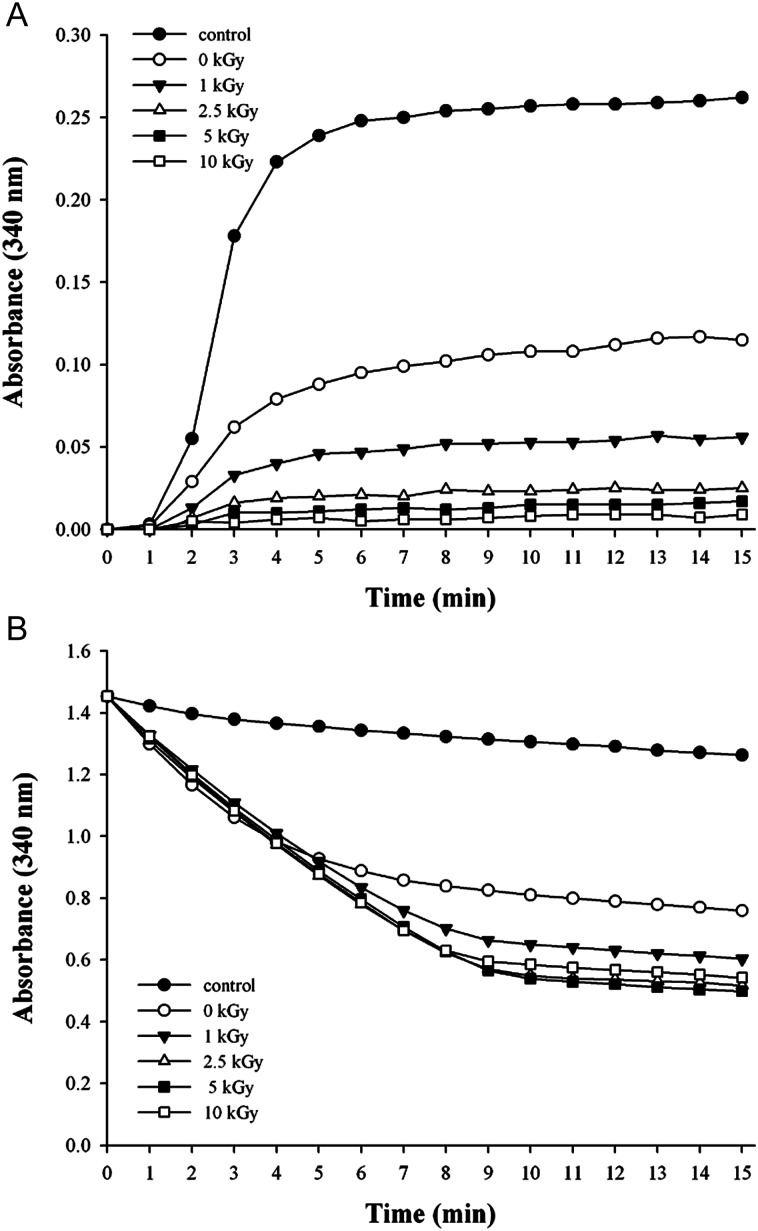
Effect of proton irradiation on enzymatic activities of PaPrx. (**A**) Chaperone activities of non-irradiated (0 kGy) and irradiated PaPrx proteins were measured using the aggregation of malate dehydrogenase (MDH) at 43°C at molar ratios of 1 vs 1 (MDH vs PaPrx). The control was measured in the absence of PaPrx protein. Data are the means of at least three independent experiments. (**B**) Peroxidase activities of recombinant PaPrx proteins measured with the yeast Trx system (3 μM yeast Trx, and 1.5 μM yeast TR) and NADPH at 340 nm. The peroxidase activities of irradiated PaPrx proteins were compared with those of non-irradiated PaPrx (0 kGy). The control indicates the peroxidase activity in the Trx system in the absence of PaPrx protein.

PaPrx, a typical 2-cysteine peroxiredoxin existing in *P. aeruginosa* PAO1, has dual functions such as a peroxidase and a molecular chaperone [25]. In this study, we first examined the molecular chaperone and peroxidase activities in order to investigate the effects of proton irradiation on the dual functions of PaPrx. The PaPrx protein was exposed to several doses of proton irradiation (1, 2.5, 5 and 10 kGy). Its chaperone activity was dramatically increased by proton irradiation compared to that of non-irradiated PaPrx. In particular, the aggregation of the MDH substrate could be completely protected at 10 kGy of proton irradiation, indicating chaperone activity about 4–5-fold stronger than that of non-irradiated PaPrx (Fig. 2A). In addition, proton irradiation had little effect on, or slightly increased, the peroxidase activity of PaPrx (Fig. 2B). In an earlier study, we found that the chaperone activity of PaPrx was greatly increased (about 3–4-fold) by gamma irradiation, but its peroxidase activity was dramatically decreased to as little as that of a control sample [17]. Interestingly, however, we found that the proton-irradiated PaPrx showed greatly increased chaperone activity together with slightly increased peroxidase activity. These changes in the dual enzymatic functions of PaPrx suggest that proton-irradiated PaPrx differed somewhat from the gamma-ray- and electron-beam-irradiated proteins. Therefore, we investigated the structural modification and properties of proton-irradiated PaPrx.

### Effects of proton irradiation on the structure and properties of PaPrx

**Fig. 3.**
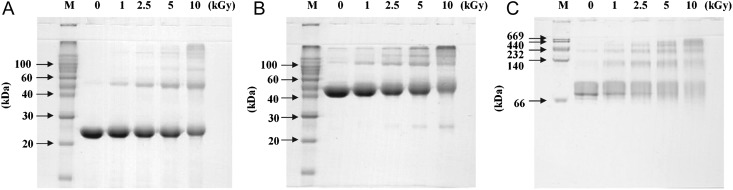
PAGE patterns of PaPrx irradiated under different conditions. Proton-irradiated PaPrx proteins were separated by 12% SDS-PAGE under (**A**) reducing conditions, (**B**) non-reducing conditions, or by (**C**) 8% native PAGE under native conditions. Proteins were stained with Coomassie Brilliant Blue R-250. Lane M shows the protein molecular weight markers.

To determine the effect of proton irradiation on PaPrx protein structure, we performed various analyses such as PAGE analysis, SEC, and CD spectroscopy. Under reducing conditions, PaPrx protein showed dose-dependent formation of oligomeric and HMW complexes, produced by chemical bonds like covalent bonds; fragmentation also occurred under high proton irradiation of about 5 kGy (Fig. 3A). However, the image of a non-reducing PAGE gel indicated that the intensity of the dimeric form, which is a characteristic of typical 2-Cys Prx proteins, was significantly decreased by proton irradiation, and the intensity of monomeric or oligomeric bands of PaPrx was increased (Fig. 3B). In addition, native PAGE analysis of proton-irradiated PaPrx revealed dose-dependent structural alterations, such as fragmentation and formation of structures of various molecular weights (Fig. 3C). In the PAGE analysis, exposed PaPrx proteins exhibited both the formation of HMW complexes and fragmentation under several doses of proton irradiation. HMW complexes or aggregates might be formed under proton irradiation by the generation of cross-linking, electrostatic and hydrophobic interaction, and disulfide bonds [20, 32].

**Fig. 4.**
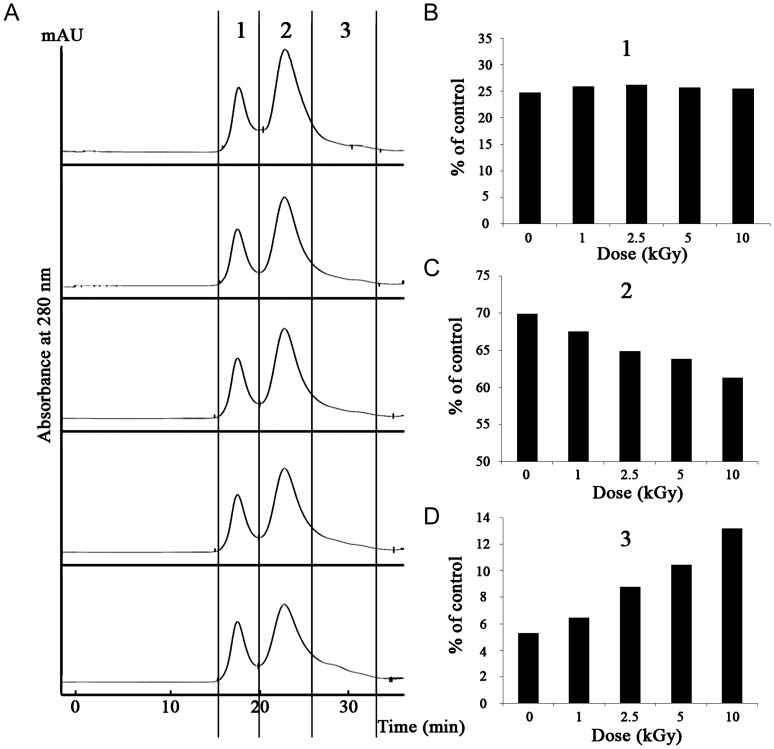
SEC profiles of non-irradiated and irradiated PaPrx proteins. (**A**) Separation of non-irradiated and proton-irradiated proteins. SEC patterns of PaPrx proteins were divided into three sections: (**B**) HMW complexes, (**C**) LMW structures, and (**D**) fragments.

The SEC pattern of non-irradiated PaPrx was divided into three sections representing HMW complexes, LMW structures, and fragments (Fig. 4A). In Fig. 4, when exposed to proton irradiation, the HMW complexes increased slightly (∼3%) compared to non-irradiated PaPrx, whereas the areas of the LMW and fragment sections changed in opposite directions; i.e. the area of the LMW section (ranging from dimer to decamer with a mass of about 40–200 kDa) decreased gradually with increasing proton irradiation dose, but the area of the fragment section exhibited an increase corresponding to fragments with a mass below ∼20 kDa. Proton irradiation caused dose-dependent enhancement of the amount of products with very low molecular mass. This structural modification of PaPrx by proton irradiation was in good agreement with the PAGE results in Fig. 3.

**Fig. 5.**
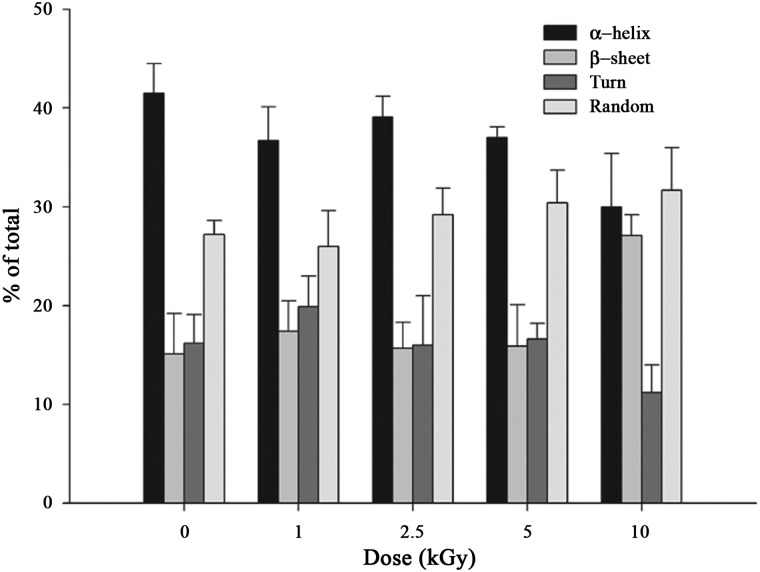
Secondary structural changes in PaPrx due to proton irradiation. The comparison of the secondary structure index values (%) was based on the far UV-CD spectra of PaPrx under different proton irradiation doses. Data are the means of at least three independent experiments.

Far-UV CD spectra were used to investigate the secondary structural elements of proton-irradiated and non-irradiated PaPrx proteins. Earlier, we found that the secondary structural elements were affected by gamma-ray or electron-beam irradiation [17, 18]. The α-helix content of irradiated proteins decreased and the β-sheet content increased compared with those of non-irradiated proteins [17, 18]. However, the secondary structural elements, such as the α-helices, β-sheets, turns and random coils, were unaffected by exposure to proton irradiation of 0–10 kGy (Fig. 5). This result suggests that proton irradiation might generate a new protein that has relatively high chaperone and peroxidase activities.

**Fig. 6.**
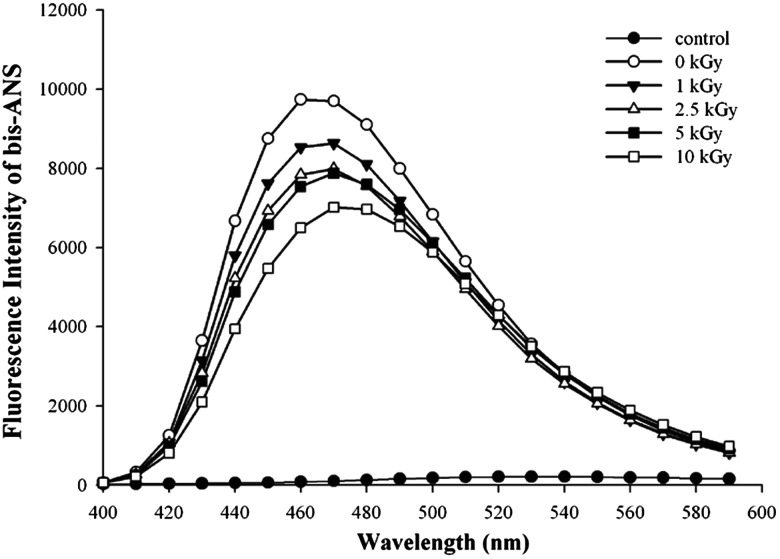
Hydrophobicity change in PaPrx after proton irradiation. Fluorescence spectra of 40 µM bis-ANS bound to 30 µg of each irradiated PaPrx protein. The control was measured in the absence of PaPrx protein. Data are the means of at least three independent experiments.

The chaperone activity of proteins is generally closely related to their hydrophobicity [33]. We compared the hydrophobicity of non-irradiated and proton-irradiated PaPrx by measuring the binding of the bis-ANS fluorophore, which is widely used as a probe in the detection of hydrophobic regions on a protein surface [31, 34]. However, this result (Fig. 6) showed that the hydrophobicity of the proton-irradiated proteins decreased with increasing irradiation dose. Reddy *et al.* [35] noted several instances in which the correlation between hydrophobicity and a protein's chaperone-like activity was paradoxical. They described numerous cases in which hydrophobicity alone does not account for the chaperone-like activity of α-crystallin. These include variations in the oligomeric size/state, subunit exchange, variations in the quaternary structure and stability of α-crystallin, and ionic interactions between the chaperone and the substrate. Finally, several factors seem to influence the chaperone-like activity; hydrophobicity may be one of these factors but not the predominant one. Further, the chaperoning function and mechanism may vary depending on the substrates and other prevailing conditions [35].

## DISCUSSION

Interest has increased in the development of industrially important protective enzymes that have increased resistance to inactivation and aggregation. In particular, enzyme engineering by site-directed mutagenesis and immobilized chaperone proteins, such as the heat shock protein (Hsp70), exhibit some potential for the stabilization and re-activation of enzymes [36, 37]. We attempted to increase the enzyme activity of Prx proteins using proton irradiation. The 2-Cys Prx proteins are members of a ubiquitous family of peroxidases that exhibit antioxidant activity as peroxidases and molecular chaperones and participate in redox-sensitive signaling [8, 13, 38]. Most 2-Cys Prxs form condition-dependent oligomeric structures, although the physiological relevance of their association or dissociation is unclear [8, 12, 13]. This work is a basic study that may facilitate the design of a protocol for increasing their enzyme activity. The results suggest that a radiation-modification protocol can be optimized on the basis of the structural and functional changes in PaPrx due to proton irradiation.

First, we examined the molecular-chaperone and peroxidase activities in order to investigate the effects of proton irradiation on the PaPrx functions. The chaperone activity of PaPrx is dramatically increased and the peroxidase activity is slightly increased by proton irradiation (Fig. 2), which differs greatly from the results of other methods, such as gamma-ray irradiation or oxidative stress. Second, we analyzed the structural changes due to proton irradiation. PAGE analysis demonstrated that proton-irradiated PaPrx proteins exhibited simultaneous HMW complex formation and fragmentation (Fig. 3), which was confirmed by the SEC patterns (Fig. 4). Finally, the secondary structural elements of PaPrx were not affected by exposure to proton irradiation at 0–10 kGy (Fig. 5). Previously, we applied functional switching of PaPrx proteins using gamma rays or electron beams as irradiation sources. We found that the irradiated protein exhibited increased chaperone activity but decreased peroxidase activity; their α-helix content decreased and β-sheet content increased compared with those of non-irradiated proteins [17, 18].

Generally, when a protein is exposed to heat stress, it is damaged or changed, or the molecular structure is denatured. The 2-Cys Prx proteins have dual functions as both a peroxidase and chaperone, and functional changes in PaPrx are closely associated with structural changes. Heat stress is a key factor in structural and functional change in the 2-Cys Prxs, which exhibit increased chaperone activity and changes in structure from LMW to HMW complexes. During proton irradiation of the order of kGy, thermal effects cannot be completely ruled out. The incident proton energy, 14.8 MeV, was reduced to 11.0 MeV, and the energy lost was supplied to the sample; on average, 0.05 J/sec of power was transferred to the sample. For proton irradiation at 10 kGy, the energy transferred to a 314 mg sample was calculated to be 3.14 J, and the calculated increase in the sample temperature was 2.75°C, assuming no thermal convection or conduction. Thus, the sample temperature can increase to 28°C from room temperature (25°C). Therefore, PaPrx proteins were not affected by heat during proton irradiation at up to 10 kGy.

The difference in effect produced by proton beams compared with gamma-rays/electron beams on peroxidase activity of PaPrx can be partly explained as due to the difference in their radiolysis products of water. Low-LET irradiations like gamma-rays are known to exert their damaging effects on bio-molecules mainly by indirect effect whereas, high-LET irradiations are damaging mainly due to direct effect. With high-LET irradiations, the radical yields decrease (e_aq_^−^, •H and •OH) whereas molecular product (H_2_O_2_, O_2_ and H_2_) yields increase due to radical–radical reactions [39]. A protein is more susceptible to oxidation/damage by free radicals, especially •OH, than by molecular oxidizing species including H_2_O_2_ and O_2_ [40]. It may be for this reason that not much loss of peroxidase activity of PaPrx was observed under proton-beam treatment, since free radicals like •OH were produced in higher concentrations during gamma-ray and electron beam treatments, and led to decrease in the peroxidase activity of PaPrx.

## CONCLUSION

This is the first study to report improvements in the enzymatic dual functions of a 2-Cys Prx. In particular, our results differed from those of studies using gamma-ray or electron-beam irradiation, and demonstrated that peroxidase activity was not reduced but maintained after proton irradiation at up to 10 kGy. Therefore, proton irradiation is a promising method for maximizing the dual functions of PaPrx. These findings are expected to be useful in various protein engineering applications and industrial processes.

## FUNDING

This project was supported by the Nuclear R&D Program of the Ministry of Science and Technology, Republic of Korea.
